# Learning to read alters cortico-subcortical cross-talk in the visual system of illiterates

**DOI:** 10.1126/sciadv.1602612

**Published:** 2017-05-24

**Authors:** Michael A. Skeide, Uttam Kumar, Ramesh K. Mishra, Viveka N. Tripathi, Anupam Guleria, Jay P. Singh, Frank Eisner, Falk Huettig

**Affiliations:** 1 Department of Neuropsychology, Max Planck Institute for Human Cognitive and Brain Sciences, Stephanstrasse 1a, 04103 Leipzig, Germany.; 2 Centre of Biomedical Research, Raibareli Road, 226014 Lucknow, Uttar Pradesh, India.; 3 University of Hyderabad, Prof. C.R. Rao Road, Gachibowli, 500046 Hyderabad, Telangana, India.; 4 Centre for Behavioural and Cognitive Sciences, University of Allahabad, University Road, Old Katra, 211002 Allahabad, Uttar Pradesh, India.; 5 Department of Psychology, University of Allahabad, 211002 Allahabad, Uttar Pradesh, India.; 6 Donders Institute, Radboud University, Montessorilaan 3, 6525 HR Nijmegen, Netherlands.; 7 Psychology of Language Department, Max Planck Institute for Psycholinguistics, Wundtlaan 1, 6525 XD Nijmegen, Netherlands.

**Keywords:** reading, illiterates, dyslexia, resting-state fMRI, functional connectivity, visual system, superior colliculus, pulvinar nucleus

## Abstract

Learning to read is known to result in a reorganization of the developing cerebral cortex. In this longitudinal resting-state functional magnetic resonance imaging study in illiterate adults, we show that only 6 months of literacy training can lead to neuroplastic changes in the mature brain. We observed that literacy-induced neuroplasticity is not confined to the cortex but increases the functional connectivity between the occipital lobe and subcortical areas in the midbrain and the thalamus. Individual rates of connectivity increase were significantly related to the individual decoding skill gains. These findings crucially complement current neurobiological concepts of normal and impaired literacy acquisition.

## Teaser

As little as 6 months of literacy instruction leads to neuroplastic changes in deep structures of the brain of illiterate adults.

## INTRODUCTION

Learning to read is a profound cultural experience requiring systematic instruction and intensive practice over months or years (*1*). Yet, hemodynamic brain activity induced by perceiving printed words changes after only a few weeks of training letter-sound links (*2*). Enhanced functional selectivity to print emerges in parts of the visual system, that is, the bilateral occipital cortices (*3*), and in a multimodal symbol processing region located in the left temporo-occipital fusiform cortex (*2*, *4*, *5*). These findings have revealed the important insight that literacy-related learning triggers cognitive adaptation mechanisms manifesting themselves in increased blood oxygen level–dependent (BOLD) responses during print processing tasks (*6*, *7*). However, it remains elusive whether reading acquisition also leads to an intrinsic functional reorganization of neural circuits.

Here, we used resting-state functional magnetic resonance imaging (fMRI) as a measure of spontaneous neuronal activity to capture the impact of reading acquisition on the functional connectome (*8*). In a controlled longitudinal intervention study, we taught 21 illiterate Hindi-speaking adults how to read Devanagari script for 6 months. The goal was to compare the changes in resting-state fMRI data before and after learning of the sample taught to read with those of a sample of nine Hindi-speaking illiterates who did not undergo such instruction. Participants were recruited from the same societal community in two villages of a rural area near the city of Lucknow in North India and matched for the most relevant cognitive, demographic, and socioeconomic variables.

Given that becoming literate goes along with widely distributed modulations of cortical responses to print, we assumed that the effects of our intervention could be best captured with a two-step procedure. First, we performed an unbiased network centrality analysis to explore functional connectivity between each voxel and all other voxels of the brain without predefining seed regions. The cluster of the most strongly connected voxels was then used as a post hoc seed region to identify the specific network driving the global change in functional connectivity.

## RESULTS

### Behavioral effects of practicing Devanagari script on letter knowledge and word-reading skills

The behavioral effectiveness of the literacy instruction was reflected in significant group (reading-trained individuals versus untrained illiterates) by time (before versus after intervention) interactions of letter knowledge [*F*_1,28_ = 17.80, *P* < 0.001, η^2^ = 0.39; 2 × 2 mixed analysis of variance (ANOVA)] and word reading (*F*_1,28_ = 15.96, *P* < 0.001, η^2^ = 0.36; 2 × 2 mixed ANOVA). Both interactions were driven by significant improvements of the trained group (letter knowledge: *z* = 4.20, *P* < 0.001, *r* = 0.65; word reading: *z* = 3.83, *P* < 0.001, *r* = 0.59; Wilcoxon signed-rank tests) that were not observed in the untrained group (letter knowledge: *z* = 0.41, *P* = 0.684; word reading: *z* = 0.37, *P* = 0.715; Wilcoxon signed-rank tests) (Table 1).

**Table 1:** Participant demographic information and behavioral performance.

	**Trained group**	**Untrained group**	**Group difference**
*n*	21	9	—
Age (years)	31.57 ± 4.90*	31.78 ± 5.47*	*z* = 0.21, *P* = 0.837
Gender (female/male)	20/1	8/1	—
Monthly income (Rupees)	2313.50 ± 629.15*	2500 ± 433.01*	*z* = 0.96, *P* = 0.375
Literate family members	2.95 ± 1.54*	2.86 ± 1.46*	*z* = 0, *P* = 1
Raven test	13.29 ± 2.67*^†^	11.67 ± 2.60*^†^	*z* = 1.42, *P* = 0.164
Letter knowledge pretest	10.38 ± 12.50*^‡^	7.22 ± 10.12*^‡^	*z* = 0.98, *P* = 0.341
Letter knowledge posttest	33.81 ± 7.11*^‡^	5.44 ± 9.84*^‡^	*z* = 4.21, *P* < 0.001
Word reading pretest	0.57 ± 1.57*^‡^	1.56 ± 2.65*^‡^	*z* = 1.41, *P* = 0.301
Word reading posttest	7.10 ± 8.53*^‡^	1.56 ± 2.35*^‡^	*z* = 2.61, *P* = 0.009
Days between tests	189.76 ± 22.74*	171.22 ± 63.85*	*z* = 1.31, *P* = 0.193

*Mean ± SD.

†Raven test raw scores (maximum 60 points).

‡Raw scores.

### Resting-state network centrality changes in the bilateral pulvinar nuclei and the right superior colliculus

**Fig. 1:**
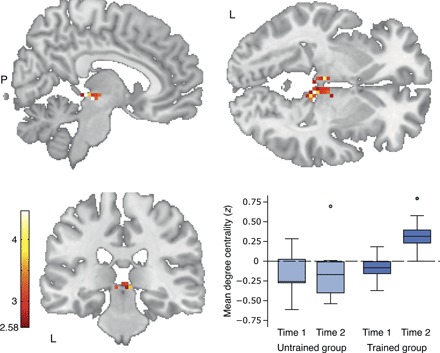
Learning to read modifies subcortical network centrality. Whole-brain degree centrality map thresholded at *z* = 2.58 (*P* < 0.005, corrected for cluster size) with corresponding color bar indicating the range of *z* scores. The effect of literacy instruction is depicted as a group (reading-trained individuals versus untrained illiterates) by time (before versus after intervention) interaction. The significant cluster stretches from the right superior colliculus of the brainstem (MNI coordinates: +6, −30, −3) to the bilateral pulvinar nuclei of the thalamus (MNI coordinates: +6, −18, −3; −6, −21, −3). The box plot resolves the interaction by separately showing the individual mean *z* values for each factor level. Mean degree centrality values of the untrained group did not differ significantly from zero (time 1: *t*_1,8_ = 1.76, *P* = 0.116; time 2: *t*_1,8_ = 1.10, *P* = 0.302; one-sample *t* tests).

Initially, we investigated in a voxel-wise fashion at the whole-brain level whether the experience of becoming literate modifies network nodes of spontaneous hemodynamic activity. Therefore, we compared training-related differences in the degree centrality of BOLD signals between the groups (*9*). A significant group by time interaction (*t*_max_ = 4.17, *P* < 0.005, corrected for cluster size) was found in a single coherent cluster (*k* = 35 voxels; voxel size 3 × 3 × 3 mm^3^) extending from the right superior colliculus of the brainstem [MNI (Montreal Neurological Institute) coordinates: +6, −30, −3] to the bilateral pulvinar nuclei of the thalamus (MNI coordinates: +6, −18, −3; −6, −21, −3) (Fig. 1). This interaction was characterized by a significant mean degree centrality increase in the trained group (*t*_1,20_ = 8.55, *P* < 0.001, *d* = 1.31; paired *t* test) that did not appear in the untrained group, which remained at the baseline level (*t*_1,8_ = 0.14, *P* = 0.893; paired *t* test) (Fig. 1). To establish the reliability of the training-induced increase in subcortical network centrality, we performed a confirmatory leave-one-out cross-validation analysis. A linear binary support vector machine classification revealed that the experimental and control groups are not statistically distinguishable before the training (accuracy, 54.76%; *P* = 0.272), but do show a statistically significant difference after the training (accuracy, 76.98%; *P* = 0.017).

### Increasing temporal coupling of spontaneous BOLD activity in the subcortical visual nuclei and the visual cortex

**Fig. 2:**
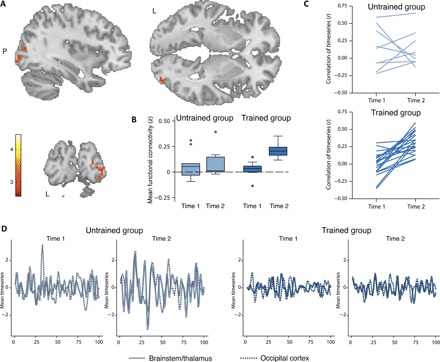
Learning to read strengthens cortico-subcortical functional connectivity. (**A**) Voxel-wise functional connectivity map derived from seeding in the significant degree centrality cluster. The image is thresholded at *z* = 2.58 (*P* < 0.005, corrected for cluster size). The color bar indicates the range of *z* scores. Becoming literate goes along with increased coupling of BOLD signal time courses between mesencephalic/diencephalic visual nuclei and a single cluster spanning the areas V1, V2, V3, and V4 of the right occipital cortex (MNI coordinates: +24, −81, +15; +24, −93, +12; +33, −90, +3). (**B**) The group (reading-trained individuals versus untrained illiterates) by time (before versus after intervention) interaction becomes evident from the box plot, indicating that the functional connectivity is strongly and specifically enhanced in the group that underwent reading instruction. (**C**) Line graphs depicting the coefficients of the correlations between the hemodynamic time series separately for each individual subject, each group, and each time. (**D**) Mean time series of the BOLD signal for each group and each time.

The cluster obtained from the degree centrality analysis was then used as a seed region in a voxel-wise functional connectivity analysis (*10*). Our aim was to identify brain areas whose BOLD time courses became more strongly coupled to those of the right superior colliculus and the bilateral pulvinar nuclei as a consequence of learning to read. A significant group by time interaction (*t*_max_ = 4.45, *P* < 0.005, corrected for cluster size) emerged as a single coherent cluster in the areas V1, V2, V3, and V4 of the right occipital cortex (*k* = 48 voxels; voxel size 3 × 3 × 3 mm^3^; MNI coordinates: +24, −81, +15; +24, −93, +12; +33, −90, +3) (Fig. 2). The cortico-subcortical mean functional connectivity got significantly stronger in the group that took part in the reading program (*z* = 3.77, *P* < 0.001, *r* = 0.58; Wilcoxon signed-rank test) but not in the group that remained illiterate (*z* = 0.77, *P* = 0.441; Wilcoxon signed-rank test).

### Stronger functional coactivation in the early visual pathway and the individual gain in letter and word knowledge

Finally, we wanted to find out whether there was a relation between the detected neural alterations and the behavioral improvements at the individual level. To this end, we derived an index for the growth of brain-functional connectivity [correlation coefficient of the BOLD time courses of each of the two regions of interest (ROIs) after minus before the intervention] and two indices for the increase of literacy (letter knowledge/word-reading skills after minus before the intervention). Individual slopes of cortico-subcortical connectivity were significantly associated with improvement in letter knowledge (*r* = 0.40, *P* = 0.014; one-sided Pearson’s correlation) and with improvement in word-reading ability (*r* = 0.38, *P* = 0.018; one-sided Spearman’s rank correlation).

## DISCUSSION

We used resting-state fMRI to examine the specific effects of learning Devanagari script on the functional connectome of illiterate Hindi-speaking Indian adults within the framework of a controlled longitudinal design. Network centrality of spontaneous hemodynamic activity significantly increased with training in the bilateral pulvinar nuclei of the thalamus and the right superior colliculus of the brainstem. Moreover, BOLD signal time courses of these subcortical structures were significantly more strongly coupled with the areas V1 to V4 of the right occipital cortex after acquiring basic literacy skills. Individual gains in intrinsic functional connectivity turned out to be significantly associated with individual gains in letter identification and word-reading skills.

Currently existing neurobiological models of reading assume that literacy boosts low-level hemodynamic responses to complex visual objects in areas V1 to V4 of the occipital cortex (*6*). Here, we provide the first evidence for functional neuroplasticity in mesencephalic and diencephalic nuclei upstream of V1 as a consequence of reading acquisition. These results call for a reconceptualization of the neural basis of reading by expanding the experimental perspective from one focused solely on the cortex to one that also includes the subcortical areas associated with oculomotor control and selective visuospatial attention.

Nonhuman primate experiments on visual motion perception suggest that the superior colliculi support the initiation of saccadic eye movements (*11*). Accordingly, the observed increase in connectomic centrality of the right superior colliculus in the course of literacy training might reflect the fine-tuning of oculomotor activity necessary for guiding fixations through printed text. An explanation for the effect in the bilateral pulvinar nuclei can be derived from numerous studies in humans highlighting the central role of these thalamic structures for selectively allocating attentional resources to visual stimuli (*12*–*16*). This is in line with several independent studies suggesting a causal role of visuospatial attention skills for reading acquisition. Namely, it has been repeatedly shown in preliterate children that visuospatial skills predict reading outcome (*17*, *18*). Moreover, there is evidence that reading abilities can be improved by training with an action video game that challenges visual attention (*19*).

If interpreted in light of recent nonhuman primate work, enhanced functional connectivity between the subcortical nuclei and the right occipital cortex detected after reading intervention indicates that the pulvinar is involved in synchronizing information transmission across the visual cortex (*20*, *21*). Signal exchange between these structures is hypothesized to be located anatomically along the long-distance white matter fiber tract that directly connects them (*22*, *23*).

Literacy-driven functional modulations of the right occipital cortex were not restricted to V1 and V2, as one would expect for alphabetic writing systems (*24*), but extended into V3 and V4. This might be explained by the nature of the Devanagari script, which is visually more complex than alphabetic writing systems. Devanagari is written from left to right and used for over 100 languages other than Hindi (for example, Bengali, Nepali, and Tibetan) and by hundreds of millions of people. It is an alpha-syllabic writing system comprising the so-called *aksharas* that represent sound simultaneously at the syllable and phoneme level. Vowels and consonants are, thus, not ordered sequentially as independent letter units in words. Devanagari is similar to alphabetic writing systems in that symbols mostly convey a word’s phonology (that is, distinct units that correspond to individual phonemes rather than syllables or words). However, Devanagari is also similar to logographic writing systems (for example, Japanese, Chinese) in that it also consists of visually complex symbols that are larger than phonological units and that are indivisible in that some of the component parts (for example, diacritic signs) cannot stand alone. In line with our finding in Devanagari, fMRI effects in V3 and V4 during print processing are known from Chinese readers (*25*). Right-lateralized manifestations of functional plasticity in the occipital cortex after training reading-related decoding skills have been repeatedly found especially in comparable samples of illiterate adults reaching modest performance levels but remain to be illuminated in future studies (*3*, *4*).

Previous task-based fMRI experiments have associated the process of learning to read with increasing BOLD responses in the so-called “visual word form area” (VWFA) of the left temporo-occipital fusiform cortex (*2*, *4*). We hypothesize that the high visual processing demands arising from the complex visuospatial arrangement of Devanagari characters might have induced a strong training effect in low-level visual areas (*26*), and that the potentially more subtle effect of symbolic learning in the VWFA would not reach statistical significance. Follow-up studies combining event-related fMRI paradigms with resting-state fMRI are necessary to confirm this hypothesis. However, we did not expect to be able to identify the VWFA when seeding in subcortical nuclei of the visual pathway to examine their resting-state functional connectivity. The VWFA has been shown repeatedly to be functionally connected to the dorsal attention network and not to lower-level visual areas when examining BOLD signals at the low-frequency sampling range covered in resting-state fMRI (*27*, *28*).

Recent cross-sectional MRI studies on adults and school-age children have reinvigorated the long-standing view that functional deficits and structural disruptions of the thalamus might play a role in developmental dyslexia, the most common learning disorder characterized by severe difficulties in learning how to read and spell (*29*–*32*). Our results indicate that the functional connectivity profile of the thalamus can change substantially even after 6 months of reading instruction in adulthood. Hence, beginning readers appear to train their subcortical sensory and attentional systems intensively. Therefore, one of the core challenges for the field is to determine whether thalamic abnormalities are a potential causal factor for developmental dyslexia or just a consequence of the impoverished reading experience of dyslexic individuals. Recent behavioral work suggests that visual motion processing skills are causally related to literacy acquisition. Specifically, dyslexic individuals perform such tasks more poorly than age-matched and reading level–matched controls (*33*, *34*). This could mean that a disruption of the underlying neural pathway connecting the lateral geniculate nucleus of the thalamus with V5 might be a contributing cause of dyslexia. Whether a similar role can be ascribed to the pathway connecting the pulvinar nuclei of the thalamus with the occipital cortex must be determined in follow-up studies. In particular, longitudinal studies following preschool children are needed to disentangle physiological causes from consequences arising from impaired literacy acquisition in scripts carrying both alphabetic and logographic features (*35*).

Learning-induced changes in coupling of spontaneous functional responses support the encoding or consolidation of novel experiences (*36*–*38*). Specifically, increased connectivity of functionally distinct areas might reflect the synchronization of excitability states of different neuronal populations (*39*). Future work on animal models combining resting-state fMRI and electrophysiological recordings is needed to confirm this hypothesis.

Note that the size of the sample investigated, though comparable to recent fMRI studies of literacy acquisition (*4*), is nevertheless small. Another limitation is that the training effects of the intervention group were compared with a passive, but not an active, control group. Accordingly, it remains to be shown whether the results reported here are literacy-specific or a general effect of visual training involving intricate symbols.

In conclusion, we have shown that only 6 months of learning to read leads to massive macroscopic functional reorganization processes in the mature human brain. When becoming literate in adulthood, spontaneous hemodynamic activity of mesencephalic and diencephalic nuclei is strongly coupled with hemodynamic activity of the occipital cortex. These findings crucially complement current neural concepts of reading by suggesting that literacy experience reshapes the earliest visual computation centers even before reaching the primary visual cortex. It remains to be shown whether deficits in these subcortical structures are a consequence of the reduced literacy experience of dyslexic individuals or a potential cause of their disorder.

## MATERIALS AND METHODS

### Participants

Participants were recruited from two villages near the city of Lucknow in the northern Indian state of Uttar Pradesh as part of a study that was approved by the ethics committee of the Center of Biomedical Research, Lucknow. After giving informed consent, 51 healthy right-handed human volunteers without a known history of psychiatric disease or neurological condition took part in the reading training and in the resting-state fMRI experiment. For unknown reasons, 18 participants did not complete the scanning sessions and were therefore excluded from further analysis (see “Demographic and behavioral data” for more details). Three additional participants were disregarded because their fMRI data did not pass our quality control procedure (see “MRI data” for more details). Accordingly, 30 participants (mean age, 31.63 years; two males; Table 1) were included in the final behavioral and neural analyses. At the beginning of the study, all of them self-reported that they were never taught how to read, spell, or write and never attended school. Subsequently, they were first assessed for their actual letter (*akshara*) knowledge and word-reading skills (Table 1) and then underwent MRI scanning. Not one of them was able to read more than eight simple words at the beginning of the study. The participants were randomly assigned either to the group that received reading instruction (*n* = 28 at the beginning of the study; *n* = 21 included in the final analysis) or to the group that did not receive any instruction (*n* = 23 at the beginning of the study; *n* = 9 included in the final analysis). Final sample sizes were similar to recent fMRI studies of literacy acquisition (*4*). Group assignment was based on the following order: The first subject was assigned to the training group, the next subject to the control group, the third subject to the training group, and so on. For organizational reasons, all investigators knew the group allocation during acquisition and analysis of the data. The instructor was a professional teacher who followed the locally established method of reading instruction. During the first month of instruction, reading and writing of the 46 primary Devanagari characters were taught simultaneously. The practice of *aksharas* was followed by the practice of two-syllable words. Approximately 200 words were taught in the first month. During the second month, participants were taught to read and write simple sentences containing mostly two-syllable words. In the third month of instruction, the participants started to learn three-syllable words and continued to practice reading and writing of simple sentences. For the remaining 3 months of the program, more complex words and some basic grammar rules were taught. For example, the participants learned about the differences between nouns, pronouns, verbs, proverbs, and adjectives and also about basic rules of tense and gender. At the end of the study, that is, approximately 6 months later (mean gap, 184 days), participants were first scanned and then tested again on the same day for their *akshara* letter knowledge and word-reading skills. The pretest items (used before the intervention) and posttest items (used after the intervention) were identical. We cannot exclude the possibility that the participants—as a side effect of literacy—were more frequently exposed to complex pictures (for example, in magazines).

### Demographic and behavioral data

Participants were matched for age, gender, handedness, income, number of literate family members, and nonsymbolic intelligence (Table 1). Each variable revealed a significant result either in a Kolmogorov-Smirnov test or in a Shapiro-Wilk test for normality of distribution, so that nonparametric Mann-Whitney *U* tests were run to compare the groups. No significant differences were found for any of the variables (all *z* < 1; Table 1). The 18 excluded participants who did not complete the scanning sessions were significantly younger (*z* = 2.97, *P* = 0.003; Mann-Whitney *U* test), performed significantly better in the test of nonsymbolic intelligence (*z* = 2.17, *P* = 0.030; Mann-Whitney *U* test), and had significantly fewer literate family members (*z* = 2.54, *P* = 0.011; Mann-Whitney *U* test) compared to the included 30 participants who completed the sessions. The groups showed no significant difference either in letter knowledge (*z* = 0.47, *P* = 0.638; Mann-Whitney *U* test) or word-reading (*z* = 0.62, *P* = 0.538; Mann-Whitney *U* test) ability at the beginning of the study (see below for details regarding these measures). Information on age, income, and number of literate family members was obtained by personal interview. Right-handedness was also verified in an interview by asking the participants which hand they used for common activities (for example, drawing). Raven’s Progressive Matrices were administered to test for nonverbal abilities.

Two measures of literacy were taken, namely, letter identification (knowledge of the 46 primary Devanagari characters) and word-reading ability (knowledge of 86 words of varying syllabic complexity). The effects of literacy instruction on behavioral performance were statistically evaluated using SPSS (www.ibm.com/software/de/analytics/spss/) to calculate a 2 × 2 mixed-design ANOVA with time [test performance before the (non-)intervention versus test performance after the (non-)intervention] as a within-subjects factor and group (illiterates who underwent intervention versus illiterates who did not undergo intervention) as a between-subjects factor. ANOVA is an appropriate test here because it has been repeatedly demonstrated to yield valid results independent of the assumption of normality of data distribution (*40*, *41*), which was violated here according to the Kolmogorov-Smirnov and Shapiro-Wilk tests. Post hoc, nonparametric Wilcoxon signed-rank tests were run to compute within-subject–level changes in performance.

### MRI data

MRI examination was conducted with a 3.0-Tesla Siemens MAGNETOM Skyra (Siemens AG) whole-body magnetic resonance scanner using a 64–radio frequency–channel head coil.

For anatomical localization, T1-weighted three-dimensional magnetization-prepared rapid-acquisition gradient echo images were acquired using a pulse sequence with repetition time (TR) = 1.690 ms, echo time (TE) = 2.60 ms, inversion time (TI) = 1.100 ms, field of view (FOV) = 256 × 256, matrix size = 256 × 256 × 192, and voxel size = 1.0 × 1.0 × 1.0 mm^3^.

For resting-state fMRI (eyes closed, no active stimulation, and no explicit task), 150 T2*-weighted gradient echo echo-planar imaging volumes covering 38 slices were collected by applying a pulse sequence with TR = 2.400 ms, TE = 30 ms, FOV = 224 × 224, matrix size = 64 × 64 × 38, and voxel size = 3.5 × 3.5 × 3.0 mm^3^.

The T1 images were visually inspected for artifacts and then segmented into gray matter, white matter, and cerebrospinal fluid using the DARTEL algorithm (*42*) implemented in SPM8 (www.fil.ion.ucl.ac.uk/spm/software/spm8/). These segmentations served to create individual tissue masks and a sample-specific template in MNI space.

The fMRI data were preprocessed using the SPM8 software package (www.fil.ion.ucl.ac.uk/spm/software/spm8/) and the DPARSF toolbox (www.restfmri.net). First, the first four volumes of each scan were discarded to allow for signal equilibration. Second, the images were slice time–corrected by interpolation and resampling to the slice at the mid–time point of each TR. Third, the images were motion-corrected by realigning them to the first acquired volume. Fourth, additional motion correction was carried out by regressing out three translational and three rotational motion parameters of each volume and its preceding volume as well as the square of each of these values (*43*). Mean signals of the white matter and the cerebrospinal fluid and linear and quadratic trends were also included in this model to control for physiological noise induced by respiration and pulsating veins. Fifth, each time series was temporally bandpass-filtered (0.01 to 0.1 Hz) using an ideal rectangular filter. Sixth, the images were resampled to a spatial resolution of 3.0 × 3.0 × 3.0 mm^3^ and normalized to the sample-specific template in MNI space. Finally, the images were spatially smoothed with a 4-mm full width at half maximum Gaussian kernel, resulting in an average smoothness of 7.0 × 6.9 × 7.0 mm^3^.

To account for the confounding effect of residual head motion, we calculated the framewise displacement (FD) of each individual time series following the approach introduced by Power *et al*. (*44*). Of 33 data sets, 30 did not exceed a single-volume threshold of 0.5981 at both acquisition time points when determining the 100 volumes with the lowest FD values. The three data sets violating this criterion were removed from the further analyses. The mean FD of the least motion-distorted 100 volumes included in the final analyses was as low as 0.1036 (SD, 0.0443) for the first time point and 0.1193 (SD, 0.0600) for the second time point. Of 6000 volumes, 5394 revealed an FD < 0.2.

Whole-brain functional connectivity was computed using the degree centrality algorithm developed by Zuo *et al*. (*9*), which quantifies connectivity by counting the number of correlations of each voxel with all voxels at a threshold of *r* > 0.25 and then assigns this number as a centrality value to each voxel. This analysis was carried out in MNI space using a group-average gray matter mask of 67.441 voxels. The resulting degree centrality images were Fisher’s *r*-to-*z–*transformed and then statistically analyzed in the framework of the flexible factorial design implemented in SPM8 running a 2 × 2 mixed-design ANOVA with time [test performance before the (non-)intervention versus test performance after the (non-)intervention] as a within-subjects factor and group (illiterates who underwent intervention versus illiterates who did not undergo intervention) as a between-subjects factor. Mean FD values did not differ significantly within groups between time points (trained individuals: *z* = 0.92, *P* = 0.357; untrained illiterates: *z* = 0.53, *P* = 0.594; Wilcoxon signed-rank tests) and also not between groups (time point 1: *z* = 0.11, *P* = 0.934; time point 2: *z* = 1.15, *P* = 0.263; Mann-Whitney *U* tests) but were nevertheless entered as a nuisance covariate of interest into the ANOVA to remove any potential relations between residual head motion and the effects of interest (*45*). When testing for statistical significance, signal variance of the two groups was not assumed to be equal because group sizes were different. Accordingly, *P* values were Greenhouse-Geisser–corrected to account for potential nonsphericity of the data. Clusters, that is, connected voxels sharing at least a corner (26 voxels), were multiple-comparison–corrected by combining a type I error threshold of *P* < 0.005 with a spatial extent threshold of *P* < 0.05. The latter threshold was determined by running 10,000 iterations of a Monte Carlo simulation as implemented in the AlphaSim tool (http://afni.nimh.nih.gov/), which revealed a minimum cluster size cutoff of *k* = 35 voxels (for the 67.441 gray matter voxels). Note that the size and the smoothness of the image were determined with SPM8 rather than AlphaSim to avoid overestimating the level of significance (*46*). Individual mean *z* values of the significant clusters were extracted with the REX toolbox (https://www.nitrc.org/projects/rex/) and then plotted separately across the factor levels with SPSS to resolve the effects characterizing the interaction. A confirmatory leave-one-out cross-validation analysis was carried out by training a linear support vector machine classifier (with the goal of distinguishing group membership before and after the training) first on a random subject before quantifying its performance on the remaining data sets. In accordance with the number of subjects in the sample, this procedure was repeated 30 times, each time with a new assignment of subjects and leaving aside each of the already given observations. Classification performance was estimated by averaging the indices obtained on the different repetitions. Statistical significance was determined nonparametrically by running 10,000 iterations of a permutation test.

The seed-based voxel-wise functional connectivity analysis (*10*) was carried out by extracting the individual means of the BOLD signal time series from the significant cluster identified with the degree centrality approach and then calculating their brain-wide correlation maps, which were finally Fisher’s *r*-to-*z*–transformed. The procedure of statistical testing was identical to the procedure applied to the degree centrality maps.

Anatomical identification of all significant clusters was based on the Harvard-Oxford Subcortical Structural Atlas and the Juelich Histological Atlas implemented in FSL (*47*).

Seed-based ROI-wise functional connectivity analyses (*10*) were run by extracting the individual means of the BOLD signal time series from the two significant clusters obtained from the previous analyses and by correlating them with each other. Subsequently, the individual correlation coefficients of the BOLD time courses of each of the two ROIs obtained before the (non-)intervention were subtracted from the coefficients obtained after the (non-)intervention. In addition, the individual letter identification and word-reading test scores, respectively, acquired before the (non-)intervention were subtracted from the scores obtained after the (non-)intervention. The resulting index of increase of functional connectivity was correlated separately with the index of increase of letter identification skills (normally distributed data; Pearson’s product-moment correlation coefficient) and the index of increase of word-reading performance (not normally distributed data; Spearman’s rank correlation coefficient) in SPSS. One-sided *P* values are reported because the analyses were carried out under the a priori assumption that better literacy skills would go along with stronger functional connectivity.
